# Supplementary material for: Mechanisms governing the pioneering and redistribution capabilities of the non-classical pioneer PU.1
Source: Nat Commun. 2020 Jan 21;11:402. doi: 10.1038/s41467-019-13960-2 (PMC6972792; doi:10.1038/s41467-019-13960-2)
Supplement: Supplementary file 7 — Source data [file 41467_2019_13960_MOESM7_ESM.zip › Source_Data/FigureS2/FigureS2B_MotifScanOutput/KmeansCluster10/homerResults/motif47.similar.html]

motif47

## Information for motif47

A
C
G
T
T
A
G
C
A
G
C
T
A
T
G
C
C
T
G
A
G
A
C
T
C
T
A
G
C
T
G
A
A
C
T
G
C
T
G
A
G
C
T
A
G
A
T
C
A
C
T
G
  
Reverse Opposite:  

T
G
A
C
C
T
A
G
C
G
A
T
G
A
C
T
A
G
T
C
G
A
C
T
A
G
T
C
C
T
G
A
G
A
C
T
T
A
C
G
T
C
G
A
A
T
C
G
T
G
C
A
  

|  |  |
| --- | --- |
| p-value: | 1e-16 |
| log p-value: | -3.817e+01 |
| Information Content per bp: | 1.650 |
| Number of Target Sequences with motif | 99.0 |
| Percentage of Target Sequences with motif | 2.67% |
| Number of Background Sequences with motif | 414.8 |
| Percentage of Background Sequences with motif | 0.94% |
| Average Position of motif in Targets | 102.9 +/- 51.8bp |
| Average Position of motif in Background | 101.4 +/- 64.4bp |
| Strand Bias (log2 ratio + to - strand density) | -0.1 |
| Multiplicity (# of sites on avg that occur together) | 1.06 |
| Motif File: | file (matrix) reverse opposite |

### Similar de novo motifs found

|  |  |  |  |  |  |  |  |
| --- | --- | --- | --- | --- | --- | --- | --- |
| Rank | Match Score | Redundant Motif | P-value | log P-value | % of Targets | % of Background | Motif file |
| 1 | 0.825 | T G A C A G C T G A T C C T A G A G C T A C T G C T G A C T A G C G T A C T G A A G T C | 1e-15 | -35.622589 | 1.29% | 0.26% | motif file (matrix) |
| 2 | 0.949 | A C G T G T A C A G C T A T G C C T G A G A C T T C A G C G T A C T A G T C G A C G T A G A T C | 1e-14 | -34.345162 | 2.51% | 0.91% | motif file (matrix) |
| 3 | 0.860 | C T A G G T A C G A C T G T A C A G C T A T G C T C G A C G A T C A T G G C T A C T A G G T C A C G T A A T G C | 1e-14 | -33.412512 | 1.86% | 0.56% | motif file (matrix) |
